# BDNF improves the survival of mesenchymal stem cells cultured on pre-structured gelatin material containing strontium and calcium phosphates for bone regeneration

**DOI:** 10.3389/fbioe.2025.1596846

**Published:** 2025-07-07

**Authors:** Paul T. Itting, Benjamin Kruppke, Thomas Hanke, Vinu Vijayan, Christian Heiss, Katrin Susanne Lips

**Affiliations:** ^1^ Department of Cardiovascular and Thoracic Surgery, University Medical Center Göttingen, Georg-August-University, Göttingen, Germany; ^2^ Experimental Trauma Surgery, Justus-Liebig-University Giessen, Giessen, Germany; ^3^ Institute of Materials Science, Technische Universität Dresden, Max Bergmann Center of Biomaterials, Dresden, Germany; ^4^ Department of Trauma, Hand and Reconstructive Surgery, University Hospital of Giessen-Marburg, Giessen, Germany; ^5^ Adjunct Professor of Orthopedics, Biruni University, Istanbul, Türkiye

**Keywords:** osteoporosis, apoptosis, bone regeneration material, cytotoxicity, mesenchymal stem cells, brain-derived neurotrophic factor, strontium

## Abstract

**Introduction:**

The treatment of osteoporotic fractures is still challenging and may be improved using materials for bone regeneration, such as pre-structured gelatin with calcium and strontium phosphates (PPGC+S), combined with brain-derived neurotrophic factor (BDNF). Recently, it was shown that PPGC+S stimulates bone formation, and BDNF improves cell survival. This study aimed to analyze the combined effect of PPGC+S and BDNF on the survival of mesenchymal stem cells (MSCs) from osteoporotic and non-osteoporotic female donors.

**Methods:**

In our study, cells were seeded on PPGC+S plates with the following mineral composition of C+S of (a) 5:5, (b) 3:7, and (c) 0:10 (PPGS 0:10). Apoptosis and necrosis were measured after the addition of BDNF, followed by light microscopic analysis.

**Results:**

The application of BDNF resulted in reduced necrosis and apoptosis in all biomaterials. The lowest level of necrosis was found in the PPGC+S 5:5 group. Apoptosis was most reduced in the PPGC+S 3:7 group, although the difference compared to PPGC+S 5:5 was not statistically significant. No differences were observed between MSCs from osteoporotic and non-osteoporotic donors.

**Conclusion:**

Thus, PPGC+S 5:5 appears to be the most suitable composition for bone healing, especially when supplemented with BDNF.

## 1 Introduction

Osteoporosis is a serious health problem that affects the quality of life for many people and places a strain on the healthcare system (Pisani et al., 2016; Sambrook and Cooper, 2006). The main risk factors for developing osteoporosis are higher age and postmenopausal reduction of estrogen (Sambrook and Cooper, 2006; Kanis et al., 2019). Therefore, the prevalence and incidence of osteoporosis are expected to increase in the future due to an aging population, especially for the subgroup of postmenopausal osteoporosis in women (Sambrook and Cooper, 2006; Kanis et al., 2019; Armas and Recker, 2012). Osteoporosis arises from diminished bone mineral density and disrupted bone microstructure, leading to an increased risk for fractures (Pisani et al., 2016; Sambrook and Cooper, 2006; Kanis et al., 2019). Normal bone mineral density homeostasis is maintained by the balanced activity of both bone-forming osteoblasts and bone-resorbing osteoclasts. In osteoporosis, the increase in the activity of osteoclasts leads to alterations in bone metabolism with increased resorption of bone minerals and, therefore, poor mineralization of the bone (Chen et al., 2018; Wang et al., 2023). Current treatment options available to manage osteoporosis involve the induction of apoptosis of osteoclasts by drugs to reduce bone resorption (Kangari et al., 2020). Another treatment strategy is to stimulate bone formation. Mesenchymal stem cells (MSCs) possess the ability to differentiate into osteoblasts according to the needs of the bone microenvironment. Although the number of MSCs does not differ between osteoporotic and healthy bone, the recruitment and differentiation of MSCs are reported to be greater in healthy bone than in osteoporotic bone (Haasters et al., 2014; Atashi et al., 2015). Earlier studies depicted a decreased osteogenic potential and reduced chondrogenesis of bone marrow MSCs in osteoporotic rats compared to the healthy rats (Huang et al., 2020). Adipogenesis and the immunogenic potential of osteoporotic MSCs are elevated, which is as a major cause of impaired for poor osteoblast formation and increased production of pro-inflammatory cytokines, leading to a disrupted bone microenvironment—unlike the healthy bone MSCs. Thus, approaches to induce the migration, proliferation, and differentiation of MSCs into osteoblasts are a target in osteoporosis treatment. The primary goal of this approach will be to increase the survival rate of MSCs so that they will be able to stimulate bone formation, which helps in osteoporosis treatment.

Materials or molecules that can increase the survival rate and viability of MSCs in elderly persons can offer great support during osteoporotic fracture healing (Alves Côrtes et al., 2024; Kilian et al., 2014). Strontium is an alkaline earth metal that is being explored and used for bone health improvement in pathological conditions due to its effects in increasing bone formation compared with restricting osteoclast resorption activity (Kołodziejska et al., 2021; Marx et al., 2020; Thormann et al., 2013). Therefore, until recently, strontium ranelate was administered as a prescription drug for the treatment of osteoporotic fractures. In addition to the little-known side effects on renal function, strontium ranelate increases thromboembolism and myocardial infarction (Curtis et al., 2021). Since its usage is no longer recommended, approaches to minimize the adverse effects of strontium-based drugs are in the limelight in osteoporosis research. Materials that can sustainably release strontium molecules into the bone microenvironment are of great importance. They allow a local effect, whereas systemic administration often requires higher concentrations and, therefore, leads to increased adverse effects (Zhang et al., 2020; Kauschke et al., 2018a; Neves et al., 2017; Reginster, 2014). The biocompatible pre-structured gelatin calcium strontium phosphates (PPGC+S) materials described by Kruppke et al. (2019) and Kruppke et al. (2020) showed a positive effect on regulating the activity of osteoblasts and osteoclasts in co-culture and improved defect healing in an osteoporotic rat model. The pre-existing data imply a significant impact on bone formation *in vitro*, but due to the known effects of strontium on MSCs, the evaluation of MSCs, especially their survival, over the mentioned biomaterials is necessary to better understand the positive effects on bone formation. This is because MSCs act as the precursor of osteoblast cells, thereby having a significant impact on bone formation (Lopes et al., 2018; Wang et al., 2016). The osteoblasts serve as the crucial class of cells in the bone microenvironment, which is severely affected during osteoporosis. Thus, the induction, attraction, survival, and differentiation of MSCs contribute to the health of the fracture sites in bones and new bone formation (Armas and Recker, 2012; Lopes et al., 2018; Wang et al., 2016). According to Kruppke et al. (2020), PPGC+S improved bone defect healing by the induction of differentiation of osteoblasts in an osteoporotic animal model. These findings could be beneficial in clinical use for reestablishing a healthy bone environment during osteoporotic treatment. Additionally, functionalizing the bone regeneration material with bone-derived neurotrophic factor (BDNF) is possible as it was performed in other bone regeneration materials, as described by Kauschke et al. (2018b), and therefore, the bone regeneration process tested in this study could be a potential delivery system not only for strontium at the defect site but also for BDNF to improve fracture and defect healing.

The degradable pre-structured gelatin of PPGC+S imitates the extracellular matrix of human bone (Giraud Guille et al., 2005; Kruppke et al., 2021). Through the addition of calcium, the phosphate-pre-structured gelatin resulted in bone-like mineralization (Kruppke et al., 2019; Kruppke et al., 2021). Due to the positive effects of strontium on osteoporotic bone, portions of calcium were replaced by strontium (Kołodziejska et al., 2021; Marx et al., 2020; Baier et al., 2013). Depending on the concentration, degradation, resorbability, and the degree of osseointegration and bone formation increased (Christoffersen et al., 1997; Verberckmoes et al., 2003). By adjusting the strontium concentration of PPGC+S, it might be possible to achieve a local strontium concentration with therapeutic potential for osteoporotic bone without the adverse effects of strontium ranelate. Interestingly, the ratio of calcium to strontium in PPGC+S influences the pore size and distributed crystal structure, which also impacts bone formation (Kruppke et al., 2019; Kruppke et al., 2020; Kruppke et al., 2021; Kruppke et al., 2016). In this study, we analyzed phosphate-pre-structured gelatin with calcium-to-strontium (Ca:Sr) ratios of 5:5 (PPGC+S 5:5), 3:7 (PPGC+S 3:7), and 0:10 (PPGS).

BDNF is a secreted protein known to enhance the growth and differentiation of new neurons and synapses (Kilian et al., 2014; Verberckmoes et al., 2003; Liu et al., 2018; Quade et al., 2018). Kilian et al. (2014) demonstrated the role of BDNF in bone fracture healing, and Kauschke et al. (2018a) provided experimental evidence of BDNF in enhancing the proliferation of MSCs (Kilian et al., 2014; Kauschke et al., 2018a). Studies evaluating the indirect effect of BDNF in bone regeneration by analyzing the cross-talk between the peripheral nervous system and bone tissue network metabolism can also be found in the literature (Tao et al., 2023). Kajiya et al. (2008) described the relevance of BDNF in modulating the gene expression pattern in periodontal regeneration. However, the role of BDNF in the necrosis and apoptosis of bone MSCs in a clinical condition through a biomaterial perspective was not evaluated in earlier studies. Considering the osteoprotective nature of the biocompatible PPGC+S material, its effect in securing MSCs is of great interest as MSCs are the precursors of osteoblasts, thus playing a major role in regulating bone mineral deposition. In addition, it would be possible to integrate BDNF into PPGC+S for follow-up examination. In this study, BDNF was applied to the cell culture medium as a primary investigation.

The effect of degradable PPGC+S on MSCs was studied in the presence of BDNF in the MSC culture environment. Differences in cell death and viability in response to the material and the molecule were compared between osteoporotic and non-osteoporotic MSCs. Since cell death can occur via two different mechanisms—apoptosis and necrosis—quantification was carried out accordingly (Duvall and Wyllie, 1986; Hanks et al., 1996; Homsy, 1970). The distinction is crucial due to the differing consequences as prolonged necrosis can lead to uncontrolled inflammation (Proskuryakov and Gabai, 2010; Cummings and Schnellmann, 2021). Thus, the culture of MSCs on PPGC+S in the presence of the bioactive molecule BDNF was explored by comparing the survival rate of osteoporotic and non-osteoporotic MSCs. As the osteogenic differentiation potential of osteoporotic MSCs is reduced compared to that of MSCs derived from healthy bone, studying the cytocompatibility of the material and BDNF provides insights into their potential osteogenic effects. The study, thus, provides valuable insights into the behavior of healthy and diseased bone-derived MSCs toward novel biomaterials enriched with minerals and molecular proteins, presenting an alternative to traditional strontium-containing drugs for the management and treatment of osteoporosis.

## 2 Materials and methods

### 2.1 Harvesting and culture of MSCs

MSCs were harvested from spongy bone that remained as residual material during routine trauma surgery treatment of endoprosthesis or fracture care after written consent from every patient was received. The approval of this use of human tissue for research purposes was obtained beforehand from the local ethics commission (74/09). The samples of female patients with osteoporosis (*n* = 5) and without any chronic bone disease (*n* = 5) were included in this study. The median age of the healthy bone donors was 69 years, with a range of 28–88 years, whereas the osteoporotic donors had a median age of 76 years and ranged from 66 to 87 years. After the operation, the residual spongy bone material was washed with Hibernate A (Gibco, Life Technologies, Carlsbad, CA, United States) and digested by collagenase type I (10 μg/mL, EC 3.4.24.3; Fujifilm Wako, Neuss, Germany). Erythrocytes were lysed using a lysis buffer, and the suspension was cleaned up by filtration using a 70-µm pore filter. Finally, the cells were cultured with MesenPRO RS medium (Gibco) supplemented with 20% fetal bovine serum (FBS, Pan Biotech, Aidenbach, Germany), 1 mM GlutaMAX (Gibco), 10 μg/mL gentamicin, and 0.25 μg/mL amphotericin B (both Life Technologies) at 37°C and 5% CO_2_. The addition of 20% FBS to MesenPRO RS medium improved the primary culture conditions when MSCs were derived from primary tissue—a purpose for which the medium was not originally designed—and improved the survival of MSCs.

### 2.2 Material for bone regeneration

**FIGURE 1:**
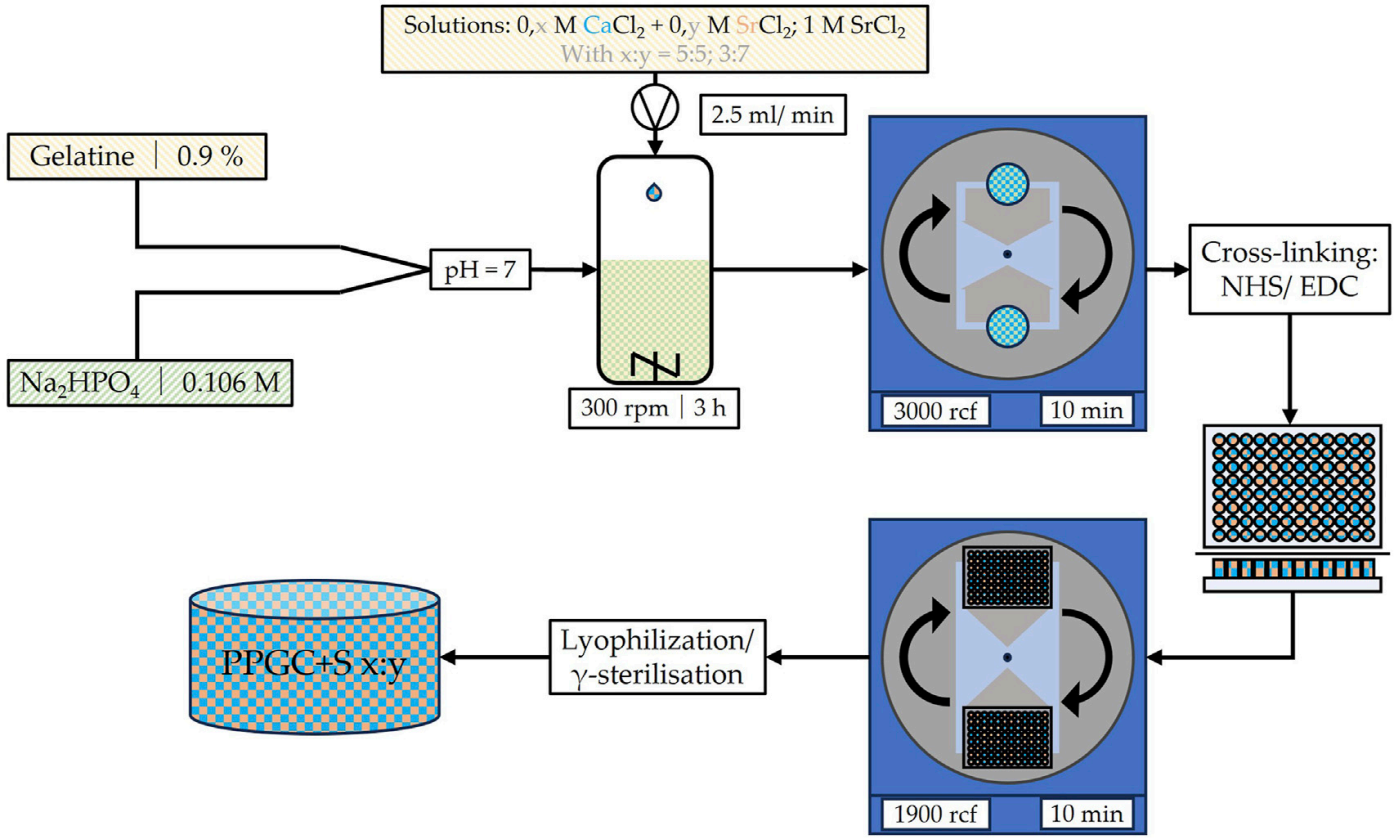
Schematic illustration of the production process for the used bone regeneration material PPGC+S as described by Kruppke et al. (2016). M, molar concentration; rpm, rounds per minute; rcf, relative centrifugal force; NHS, N-hydroxysuccinimide; EDC, 1-ethyl-3-(3-dimethylaminopropyl)-carbodiimide.

Materials for bone regeneration were produced, as shown in Figure 1 and described earlier by Kruppke et al. (2016). In brief, porcine gelatin was pre-structured by allowing 0.9% porcine gelatin (300 bloom, 20 mesh, Gelita, Eberbach, Germany) to swell for 30 min (min) in distilled water before heating the solution to 50°C in a water bath. When the temperature of 50°C was reached and the porcine gelatin was dissolved, 0.106 M disodium hydrogen phosphate (Roth, Karlsruhe, Germany) was added, the pH was adjusted to 7, and the solution was stirred constantly for 8 hours (h) before storing in a refrigerator. Afterward, the solutions of calcium chloride (Roth) and strontium chloride (Roth) were added, while the pre-structured porcine gelatin was constantly mixed on a magnetic stirrer with a hot plate. The concentrations of the calcium and strontium chloride solutions depended on the final product but were always summed as 1 M. Hence, a calcium chloride solution of 0.5 M and a strontium chloride solution of 0.5 M were used for PPGC+S 5:5. The mixed solution of calcium and strontium chloride was added to the phosphate pre-structured gelatin solution at a rate of 2.5 mL/min. The mineralization of gelatin was initiated, and the crystal particles were maintained on a stirring hot plate for 3 h to allow calcium phosphate and strontium phosphate crystals to develop their final size, structure, and morphology. After 3 h, the solution was centrifuged for 10 min at 3,000 rcf. The resulting mineral paste was then resuspended in HEPES buffer, and the pH was adjusted to 8. Then, crosslinking was induced using 1-ethyl-3-(3-dimethylaminopropyl)-carbodiimide (EDC, Sigma-Aldrich) and N-hydroxysuccinimide (NHS, Sigma-Aldrich), while the suspension was transferred to well plates and sedimented through centrifugation at 1,900 rcf for 10 min. For final crosslinking, well plates were incubated at 8°C for at least 8 h. Then, the specimens were lyophilized and sterilized by γ-ray irradiation. Figure 1 provides an insight into the production steps of the bone regeneration material used in this study.

All tested bone regeneration materials exhibit different sizes and forms of crystal structures, which result in variations in their dissolution capabilities (Kruppke et al., 2021). Particularly, the bone regeneration material with higher proportions of strontium presented with a higher release of calcium and strontium ions compared to those with fewer proportions of strontium. On the contrary, the release of phosphate ions was higher in bone regeneration materials with higher proportions of calcium. Regarding the pH-value, the experiments of Kruppke et al. (2021) showed a decrease in the pH-value over the complete experimental duration, but the absolute pH-value was higher at approximately 8 for the complete duration for all bone regeneration materials with higher strontium proportions. When assessing indirect tensile strength, it is remarkable that only bone regeneration materials with higher proportions of calcium showed an increase in the assay results. Regarding the tested bone regeneration materials in this study, PPGC+S 5:5 and PPGC+S 3:7, it should be mentioned that on day 1, PPGC+S showed higher tensile strengths than PPGC+S 5:5. However, this advantage diminished by day 14, at which point PPGC+S 5:5 showed an increase in tensile strength and outperformed PPGC+S 3:7, which presented a decrease in tensile strength over the same time-period (Kruppke et al., 2021). Because the focus of this work is on the cytotoxic capacity of these bone substitution materials, for further and more detailed physicochemical data, we refer to Kruppke et al. (2019), Kruppke et al. (2020), and Kruppke et al. (2021).

### 2.3 Live cell observation

Non-osteoporotic and osteoporotic MSCs at 100,000 cells per well were seeded on top of the bone regeneration materials already covered with the cell culture medium, either including BDNF or without it. Cells appeared to grow on the material and on the microplate surface, where they could be observed by light microscopy. Their behavior was documented on days 1, 4, and 7 of incubation using an inverse light microscope (Axiovert 10, Zeiss, Oberkochen, Germany) equipped with a Stingray F-145 camera (Allied Vision Technologies GmbH, Stadtroda, Germany).

### 2.4 Apoptosis and necrosis assay

Probes of the materials for bone regeneration were washed 3x with phosphate-buffered saline (4 mM, pH 7.4, Gibco), transferred into 24-well plates, and covered with Dulbecco’s modified Eagle medium (DMEM, Gibco) low glucose supplemented with 5% FBS, 1 mM GlutaMAX, and gentamicin/amphotericin. BDNF (40 ng/mL) was added directly before seeding MSCs (50.000/cm^2^). The medium was changed on days 1, 4, and 7, and fresh BDNF was added. Necrosis was measured on days 1, 4, and 7, whereas apoptosis was only measured on day 7 using the Cell Death Detection ELISA^PLUS^ (Roche Diagnostics GmbH, Mannheim, Germany), according to the producers’ manual. In brief, 80 µL of the kit’s incubation buffer supplemented with the kit components, anti-histone-biotin solution and anti-DNA-peroxidase, was transferred into a 96-well plate. The cells attached to a 24-well plate were centrifuged at 200 rcf for 10 min, and for the necrosis assay, 20 µL of the medium was collected. For the apoptosis assay, 600 µL of lysis buffer (kit component) was added and incubated for 30 min at room temperature. Afterward, 20 µL of the medium was collected. For the apoptosis and necrosis assay, 20 µL of the medium was transferred into the 96-well plate and incubated for 2 h on a shaker. The solution was discarded, and the wells were carefully washed with the incubation buffer. Then, 100 µL of 2,2‘-azino-bis(3-ethylbenzothiazoline-6-sulfonic acid) (ABTS, kit component) dissolved in distilled water was added. After 12 min, the reaction was terminated by the addition of 100 µL of ABTS-stop solution. The absorption (A) was measured at 405 and 490 nm using a microplate reader (BioTek, Bad Friedrichshall, Germany). The difference was calculated, followed by the determination of the enrichment factor (EF) that consists of the absorption of the experimental group divided by the absorption of the control group.

$$EF=\frac{\left(A_{experimental\;group}\;\left(405\;nm\right)-A_{experimental\;group}\;\left(490nm\right)\right)}{\left(A_{control\;group}\;\left(405\;nm\right)-A_{control\;group}\;\left(490nm\right)\right)}.$$ (1)

Equation 1 shows the calculation of the EF using absorption (A).

The enrichment factor was used for statistical analysis. Due to normalization involved in the calculation of the EF, it cannot be used to compare results between experimental days or between apoptosis and necrosis assays.

### 2.5 Statistical analysis

Before starting experimental testing, a power analysis was conducted, especially to assess the effects of BDNF, was performed. Assuming an effect size of BDNF addition between 2 and 3, with a power of 0.8 and an alpha level of 0.05, the power analysis revealed that a sample size of 3 to 6 per group was required. Statistical analysis of the results began by testing for normal distribution of the values with the Kolmogorov–Smirnov test using SPSS software (IBM, V. 24). Normally distributed values were then tested using t-test and subsequent Bonferroni–Holm correction. Non-normal distributed values were tested either using the Mann–Whitney-U test (osteoporotic versus non-osteoporotic MSC), Friedman’s two-factor variance analysis (the three different materials for bone regeneration), or the Wilcoxon-rank test (with and without BDNF). Values of p ≤ 0.05 were considered significant. The graphs were generated as box plots using SPSS software.

## 3 Results

### 3.1 Necrosis assay

#### 3.1.1 Differences in osteoporotic and non-osteoporotic MSCs

**FIGURE 2:**
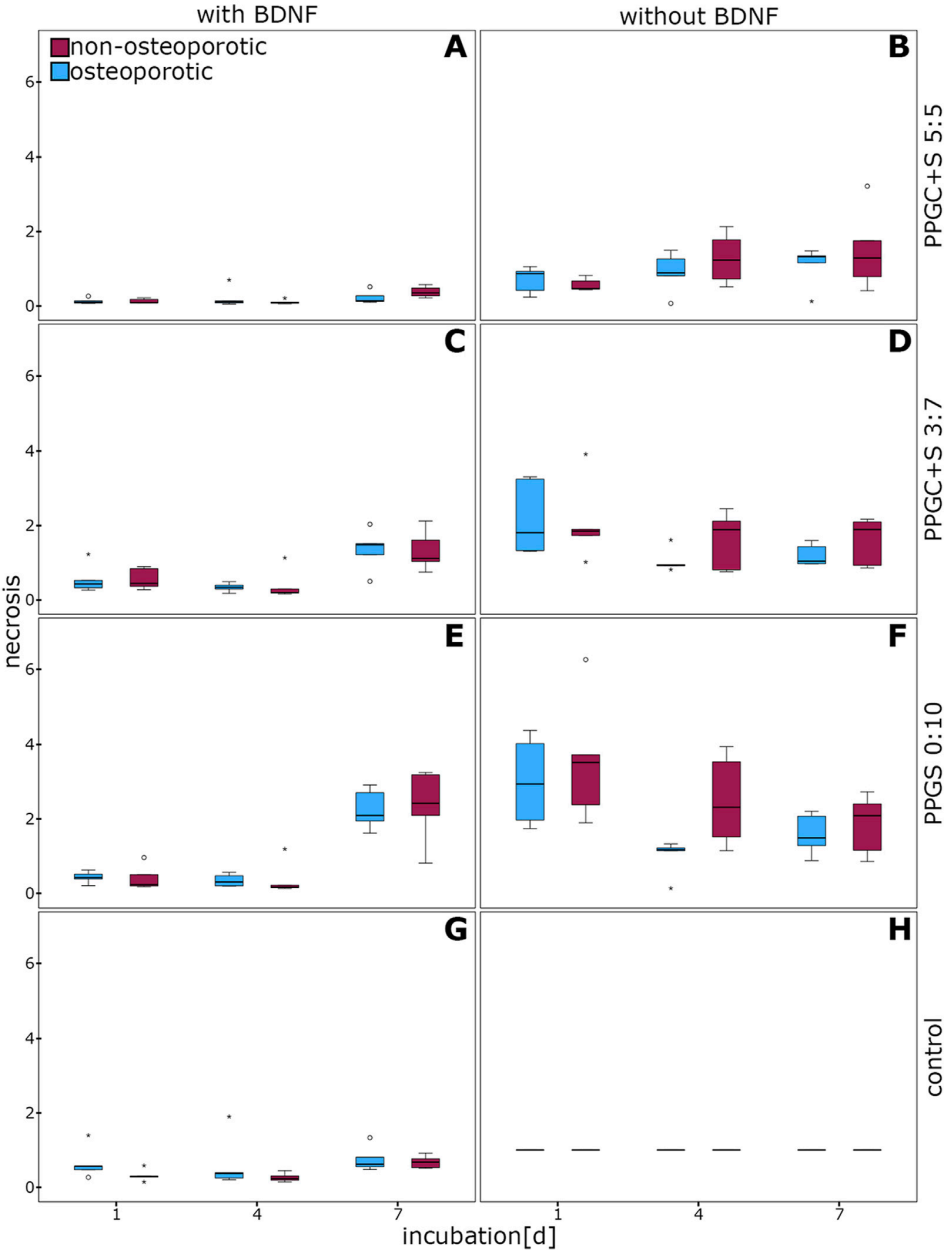
Evaluation of necrosis in MSCs. No significant differences were observed. **(A)**, **(C)**, **(E)**, and **(G)** indicates necrosis of cells with different materials with BDNF addition, while **(B)**, **(D)**, **(F)**, **(H)** indicates same materials without BDNF addition. The box plot shows the median with surrounding quartiles and interquartile range. Data outliners below three times the interquartile range are shown as dots, and those above three times the interquartile range are represented by colored stars. The x-axis displays the duration of incubation in days (d), and the y-axis displays the dimensionless enrichment factor, calculated as described above.

No significant difference in necrosis between osteoporotic and non-osteoporotic MSCs was observed (Figure 2). A tendential decrease of necrosis was measured in the osteoporotic MSCs on day 4 on PPGS 0:10 but without the supplementation of BDNF (p = 0.056; Figure 2F).

#### 3.1.2 Differences between the materials for bone regeneration

**FIGURE 3:**
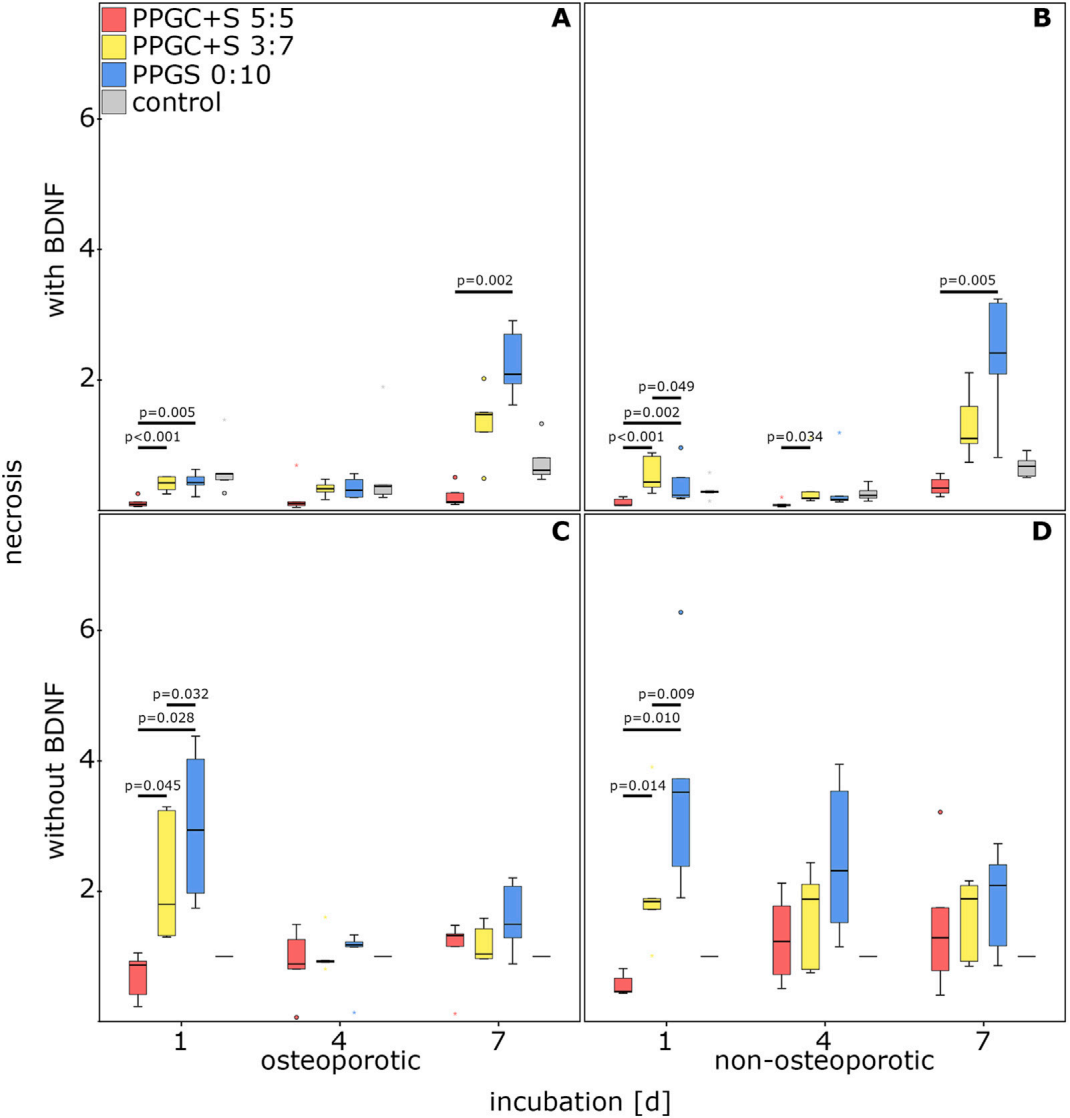
Effect of bone regeneration materials on necrosis. **(A)** and **(C)** indicate necrosis of osteoporotic MSCs with and without BDNF addition respectively. **(B)** and **(D)** indicate apoptosis of non-osteoporotic MSCs with and without BDNF addition respectively. The box plot shows the median with surrounding quartiles and interquartile range. Data outliners below three times the interquartile range are represented by dots, and those above three times the interquartile range are represented by colored stars. The x-axis displays the duration of incubation in days (d), and the y-axis displays the dimensionless enrichment factor, calculated as described above.

PPGC+S 5:5 imparted a significantly lower rate of necrosis in osteoporotic MSCs supplemented with BDNF on days 1 and 7. No significant difference was observed on day 4 (Figure 3A).

PPGC+S 5:5 also showed significantly decreased necrosis of non-osteoporotic MSCs supplemented with BDNF on day 1 compared to PPGC+S 3:7 and PPGS 0:10. In addition, on day 1, necrosis was significantly increased on PPGC+S 3:7 compared to that for PPGS 0:10. Necrosis on PPGC+S 5:5 was also significantly reduced on day 4 compared to that for PPGC+S 3:7 and on day 7 compared to that for PPGCS 0:1 (Figure 3B). Compared to the controls without the biomaterial, no significant differences were found.

Interestingly, necrosis on PPGC+S 5:5 was only significantly reduced on day 1 without BDNF. On the other days, there were no significant differences. On day 1, necrosis gradually increased: PPGC+S 5:5 < PPGC+S 3:7 < PPGS 0:10 in both types of MSCs. In control cells without the biomaterial, the necrosis level remained constant (Figures 3C, D).

#### 3.1.3 Effect of BDNF on the necrosis of MSCs

**FIGURE 4:**
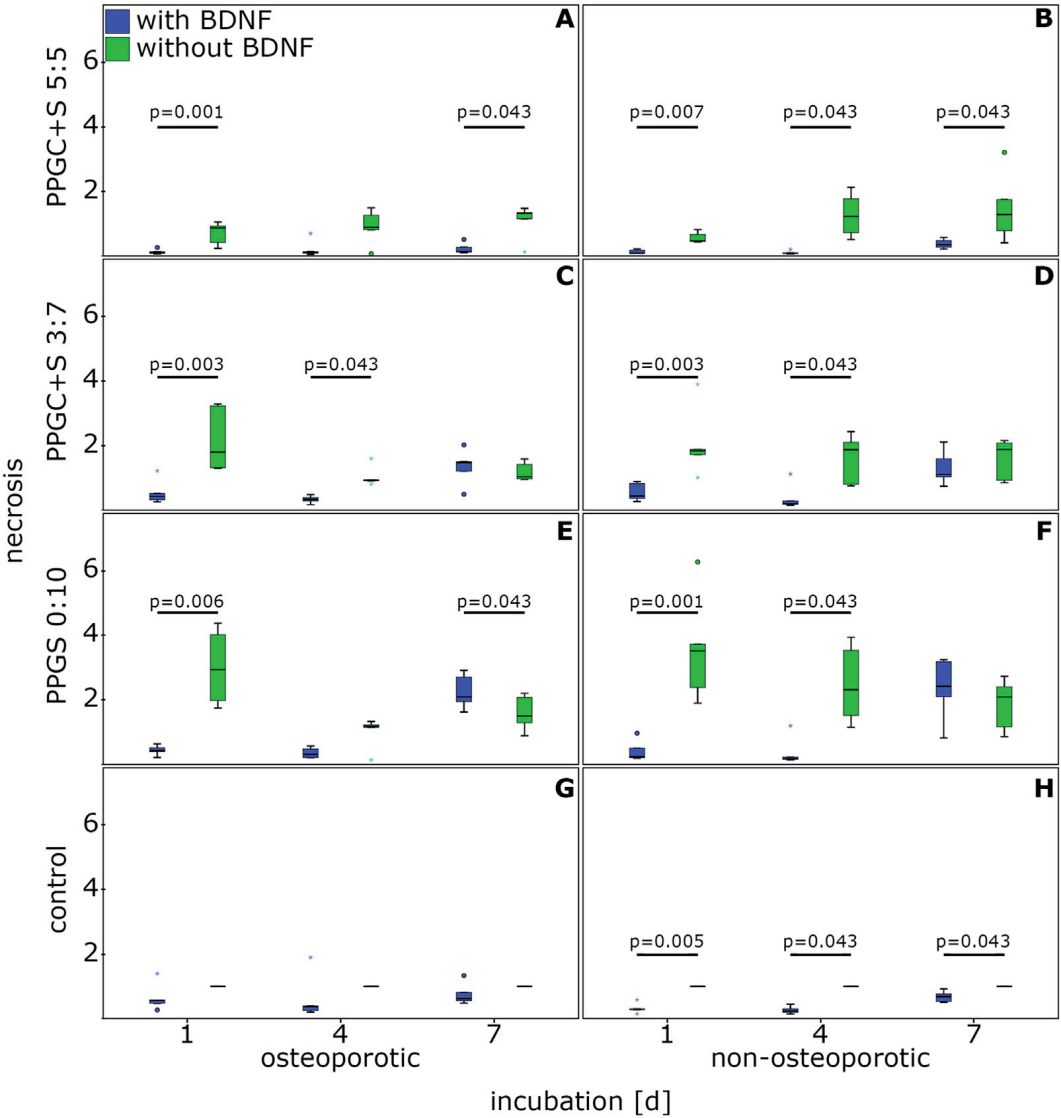
Effect of BDNF in regulating the necrosis of MSCs. **(A)**, **(C)**, **(E)**, and **(G)** indicates necrosis of osteoporotic MSC with different materials, while **(B)**, **(D)**, **(F)**, **(H)** indicates apoptosis of non-osteoporotic MSC with different materials. The box plot shows the median with surrounding quartiles and interquartile range. Data outliners below three times the interquartile range are represented by dots, and those above three times the interquartile range are represented by colored stars. The x-axis displays the duration of incubation in days (d), and the y-axis displays the dimensionless enrichment factor, calculated as described above.

The application of BDNF significantly decreased the necrosis of osteoporotic MSCs cultured with PPGC+S 5:5 on days 1 and 7 compared to MSCs cultured without BDNF (Figure 4A).

In non-osteoporotic MSCs, BDNF significantly lowered the necrosis on PPGC+S 5:5 on days 1, 4, and 7 (Figure 4B).

For PPGC+S 3:7, BDNF significantly reduced the rate of necrosis on days 1 and 4 (Figure 4C). Similarly, the presence of BDNF lowered the necrosis rate of non-osteoporotic MSCs on PPGC+S 3:7 significantly on days 1 and 4 (Figure 4D).

BDNF addition in osteoporotic MSCs caused a significant decrease in necrosis associated with PPGS 0:10 on day 1. Conversely, on day 7, the addition of BDNF presented a significantly higher necrosis than the non-addition of BDNF (Figure 4E).

Non-osteoporotic MSCs cultured with BDNF on PPGS 0:10 presented a significantly lower necrosis on days 1 and 4, but there was no significant difference on day 7 (Figure 4F).

BDNF improved the survival of non-osteoporotic control MSCs cultured without material for bone regeneration on days 1, 4, and 7, whereas no significant differences were found in the osteoporotic controls (Figures 4G, H).

### 3.2 Apoptosis assay

#### 3.2.1 Differences in osteoporotic and non-osteoporotic MSCs

**FIGURE 5:**
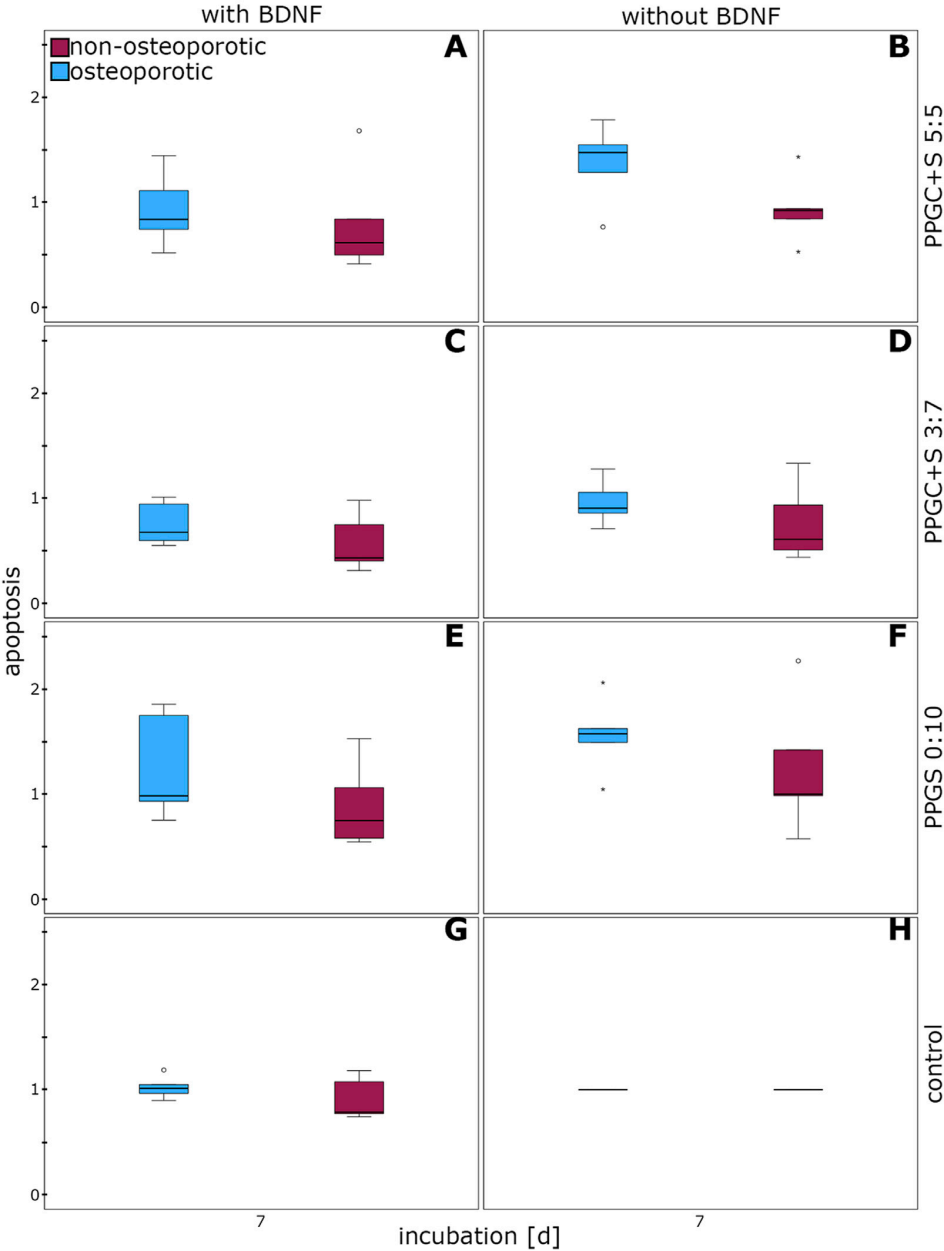
Effect of BDNF in regulating the apoptosis of MSCs. **(A)**, **(C)**, **(E)**, and **(G)** indicates apoptosis of cells with different materials with BDNF addition, while **(B)**, **(D)**, **(F)**, **(H)** indicates same materials without BDNF addition. The box plot shows the median with surrounding quartiles and interquartile range. Data outliners below three times the interquartile range are represented by dots, and those above three times the interquartile range are represented by colored stars. The x-axis displays the duration of incubation in days (d), and the y-axis displays the dimensionless enrichment factor, calculated as described above.

The apoptosis of osteoporotic MSCs did not significantly differ from that of non-osteoporotic MSCs cultured on the different bone regeneration materials, either with or without BDNF addition (Figure 5).

#### 3.2.2 Differences between the materials for bone regeneration

**FIGURE 6:**
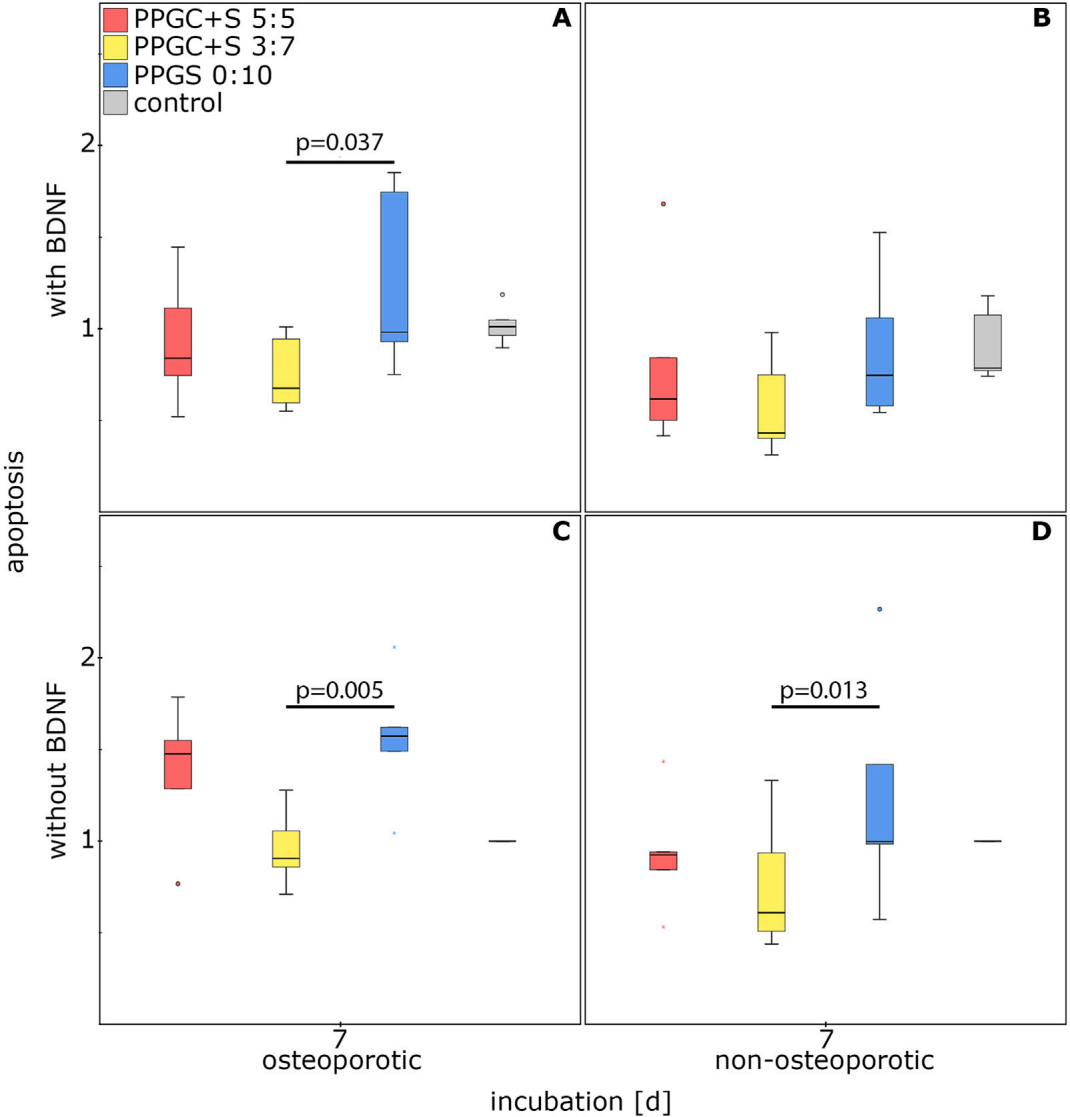
Effect of materials for bone regeneration on the apoptosis of MSCs. **(A)** and **(C)** indicate apoptosis of osteoporotic MSCs with and without BDNF addition respectively. **(B)** and **(D)** indicate apoptosis of non-osteoporotic MSCs with and without BDNF addition respectively. The box plot shows the median with surrounding quartiles and interquartile range. Data outliners below three times the interquartile range are represented by dots, and those above three times the interquartile range are represented by colored stars. The x-axis displays the duration of incubation in days (d), and the y-axis displays the dimensionless enrichment factor, calculated as described above.

Apoptosis was significantly increased in MSCs cultured on PPGS 0:10 than in MSCs cultured on PPGC+S 3:7. Neither the health status of the MSCs nor the application of BDNF changed this difference, with one exception. PPGS 0:10 did not significantly upregulate apoptosis in non-osteoporotic MSCs treated with BDNF. Thus, PPGC+S 3:7 induced the lowest apoptosis rate (Figure 6).

#### 3.2.3 Effect of BDNF on apoptosis

**FIGURE 7:**
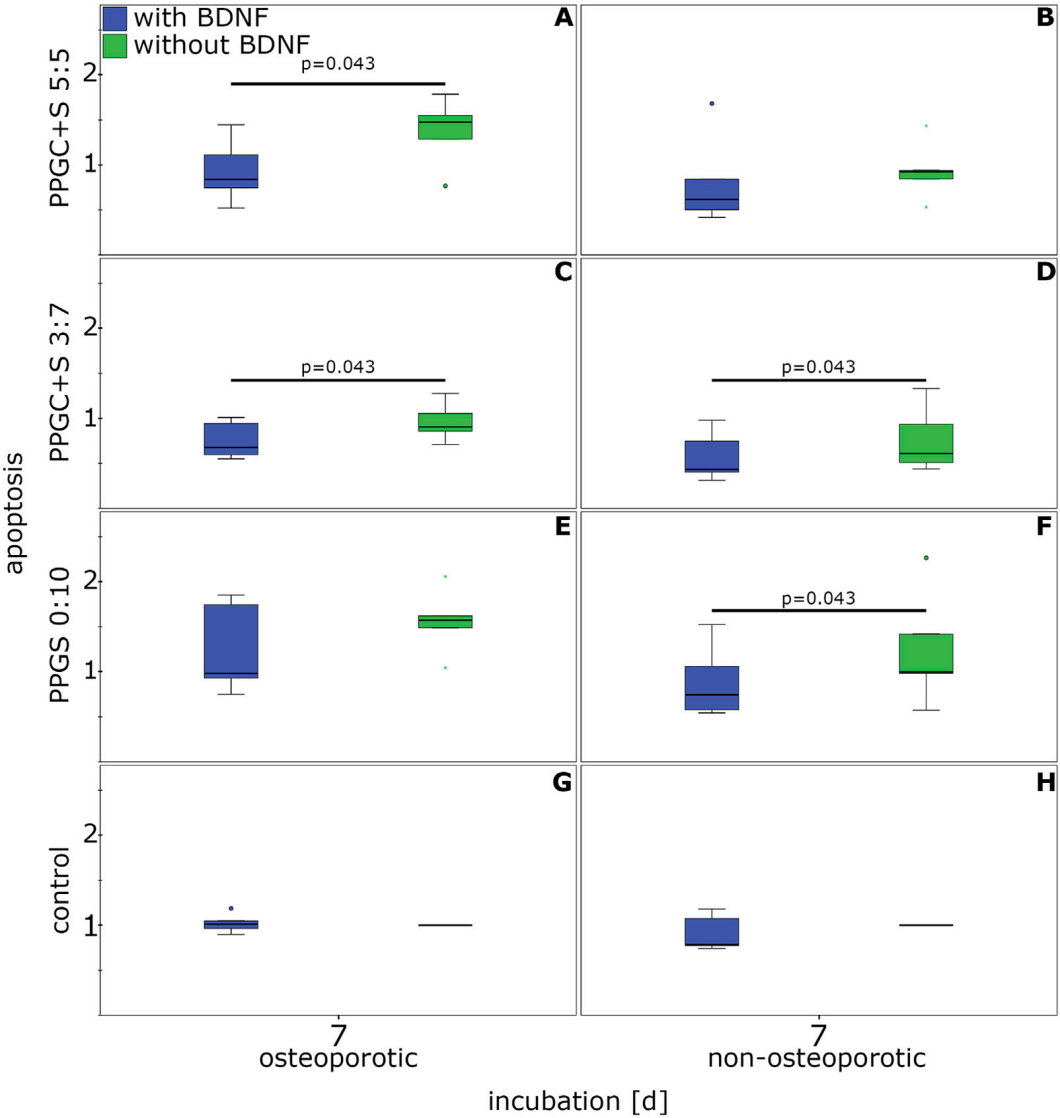
Effect of BDNF on the apoptosis of MSCs. **(A)**, **(C)**, **(E)**, and **(G)** indicates apoptosis of osteoporotic MSC with different materials, while **(B)**, **(D)**, **(F)**, **(H)** indicates apoptosis of non-osteoporotic MSC with different materials. The box plot shows the median with surrounding quartiles and interquartile range. Data outliners below three-times the interquartile range are represented by dots, and those over three interquartile ranges are represented by colored stars. X-axis displays the duration of incubation in days (d), and the y-axis displays the dimensionless enrichment factor calculated as described above.

The application of BDNF resulted in a significantly reduced apoptosis rate. This reduction was measured for osteoporotic MSCs cultured on PPGC+ 5:5 and PPGC+S 3:7 and for non-osteoporotic MSCs cultured on PPGC+S 3:7 and PPGS 0:10 (Figure 7). No significant differences were detected in the controls without materials for bone regeneration.

### 3.3 Live cell imaging

**FIGURE 8:**
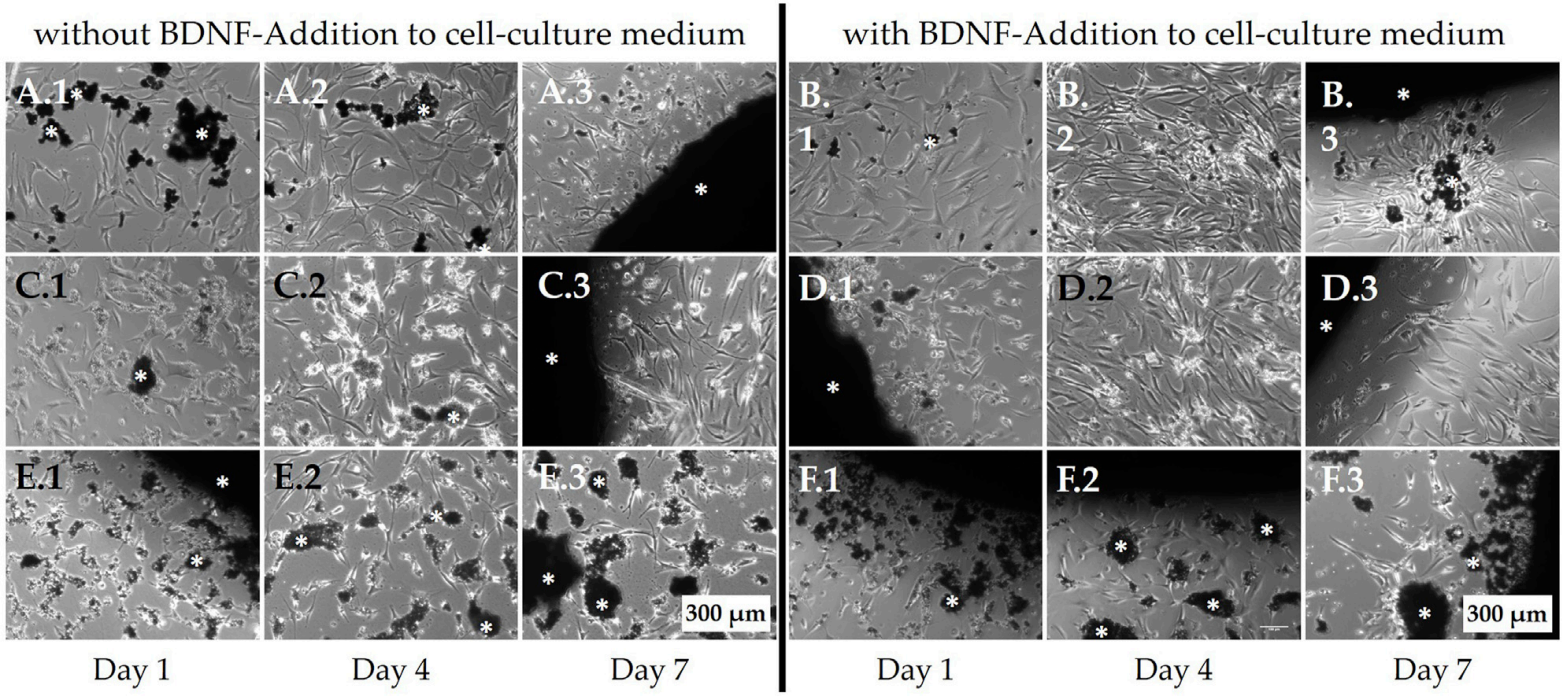
Live-cell images of non-osteoporotic MSCs. Cells were seeded on top of the bone regeneration materials and cultured in the cell culture medium. In addition to adhering to the material surface, MSCs also appeared to grow on the well plate surface adjacent to the bone regeneration materials, where they were observed using light microscopy. **(A, B)** PPGC+S 5:5, **(C, D)** PPGC+S 3:7, and **(E, F)** PPGS 0:10. **(B, D, F)** Cells after the addition of BDNF. White asterisk marks the black bone substitute material, some of which has partially disintegrated. Scale bar, 200 µm.

Non-osteoporotic MSCs (100,000 per well) were seeded on top of the bone regeneration materials but also adhered to the cell culture plate surface, allowing their behavior to be observed using live-cell light microscopy (Figure 8). The cells on PPGC+S 5:5 and PPGC+S 3:7 did not avoid the material and appeared vital even when covered with dismantled particles of the material (Figures 8A, C). By applying BDNF, a slight increase in cell number was observed on PPGC+S 5:5 and PPGC+S 3:7 (Figure 8A2 compared to B.2 and C.2 compared to D.2). In addition, cells formed more interactions with each other on PPGC+S 5.5 after the application of BDNF (Figures 8B2, B3). In the cultures with BDNF, the cell number decreased from day 4 to day 7, and sometimes, the cells showed less interaction with each other and less adhesion to the bone regeneration material (Figures 8D2, D3). On PPGS 0:10, the cells were less dense, and the cells appeared more condensed and less connected to each other (Figures 8E, F). After the addition of BDNF, the vitality of the cells slightly increased (Figures 8F.1–3). However, the cells still appeared less vital than those on PPGC+S 5:5 and PPGC+S 3:7.

**FIGURE 9:**
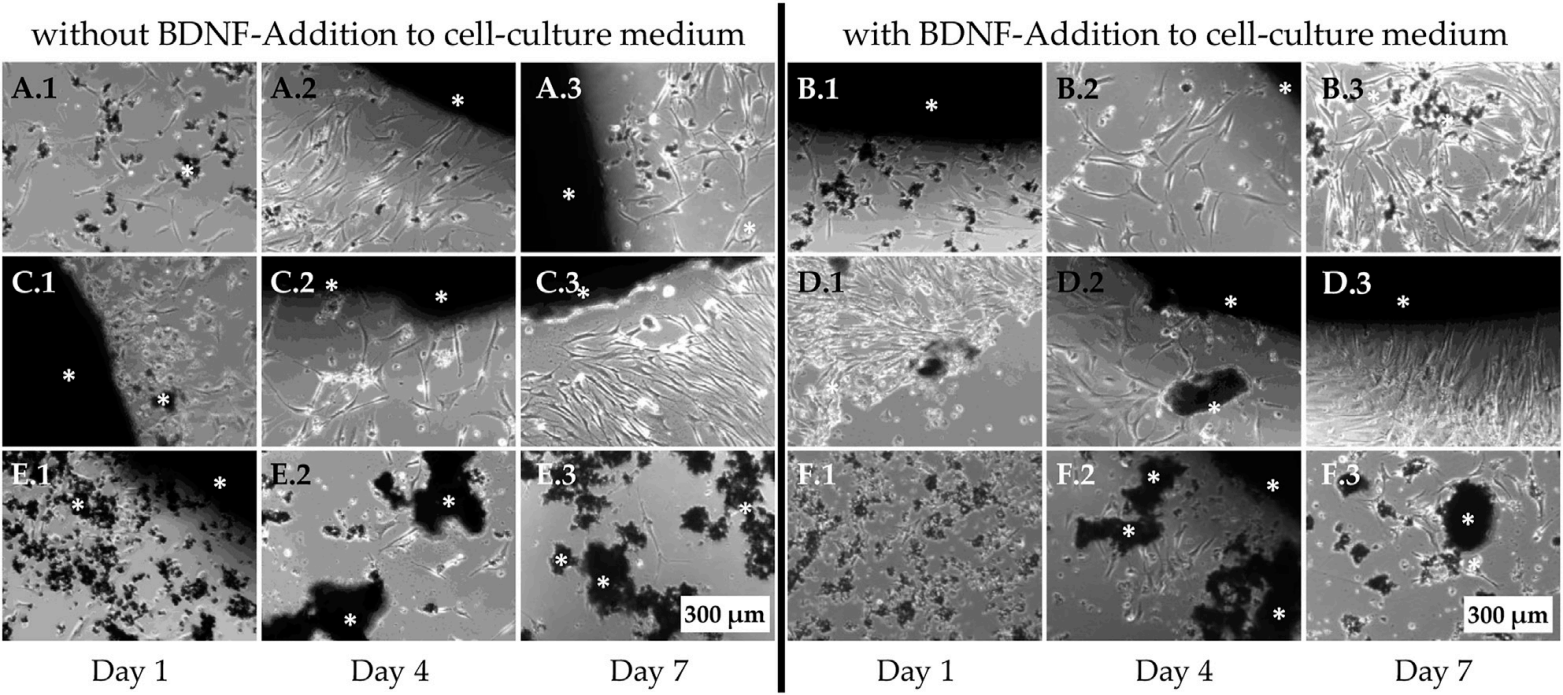
Live-cell images of osteoporotic MSCs adjacent to the bone regeneration material. **(A, B)** PPGC+S 5:5, **(C, D)** PPGC+S 3:7, and **(E, F)** PPGS 0:10. **(B, D, F)** BDNF application. White asterisk marks black bone substitute materials. Scale bar, 200 µm.

Osteoporotic MSCs that were also seeded with 100,000 cells per well revealed a remarkable decrease in cell density on day 4 in all material groups (Figure 9). As observed for non-osteoporotic MSCs, on PPGS 0:10, the cells suffered the most, and BDNF was not able to reverse this (Figures 9E, F). On PPGC+S 5:5, BDNF application led to more interaction between MSCs, whereas cell size and vitality appeared to remain constant (Figures 9B.1–3). Only the cell density seemed to vary. It appeared to decrease on day 7 without BDNF (Figure 9A3), whereas with BDNF, an increase in cell number was observed (Figure 9B3). On PPGC+S 3:7, BDNF seemed to increase the cell number (Figures 9D.1–3). The interactions, cell size, and visible signs of vitality seemed to be similar.

## 4 Discussion

This study presented the data of necrotic and apoptotic cell death of osteoporotic MSCs compared to non-osteoporotic MSCs when cultured with different materials for bone regeneration with the supplementation of BDNF. Osteoporotic MSCs are characterized by reduced osteogenic capacity and increased adipogenesis compared to MSCs derived from healthy bone, leading to poor bone formation, which can result in fractures (Pino et al., 2012). As MSCs act as the precursor of both osteoblasts and adipocytes, an increased pro-adipogenic gene expression reduces osteoblast formation in osteoporosis. Interestingly, the proliferation rate of the available MSCs remains unaffected in osteoporosis (Cassidy et al., 2024). Similarly, our study using bone substitute materials did not reveal any significant differences between osteoporotic and non-osteoporotic MSCs in terms of apoptotic or necrotic cell death. This indicates that MSCs from osteoporotic and non-osteoporotic subjects remain comparable in susceptibility toward apoptosis and necrosis. Stenderup et al. (2001) also showed that MSCs derived from osteoporotic and healthy bone did not differ in the number or proliferative capacity, but the differences were visible in the recruitment and differentiation of MSCs, with an advantage observed in non-osteoporotic MSCs (Haasters et al., 2014; Atashi et al., 2015). This work primarily involved testing the parameters of apoptosis and necrosis of MSCs as undifferentiated cells. In order to maintain consistency in the number and behavior of MSCs, they were not induced to undergo differentiation.

The behavior of the materials for bone regeneration toward MSCs was investigated by evaluating the differences in apoptotic and necrotic cell death. Materials that impair cell survival are defined as cytotoxic. Cytotoxicity can be used as a surrogate parameter for assessing the integration of a material for bone regeneration into bone tissue. In this study, we showed that the material for bone regeneration, PPGC+S 5:5, leads to lesser necrosis of MSCs compared to PPGC+S 3:7 and PPGS 0:10, independent of the addition of BDNF in both osteoporotic and non-osteoporotic MSCs. This implies that materials for bone regeneration with higher concentrations of strontium might lead to higher necrotic cell death. When comparing the results of necrotic and apoptotic cell death, it was surprising that PPGC+S 3:7 showed the lowest level of apoptotic cell death, even though the statistical difference compared to PPGC+S 5:5 was not significant.

Based on these results, it can be concluded that PPGC+S 5:5 exhibits the least toxic effects. The comparatively increased toxic behavior of PPGC+S 3:7 can be attributed to the higher concentration of added strontium, leading to a disturbed crystal structure of the material for bone regeneration, which may influence the local chemical milieu (Verberckmoes et al., 2003). Our results are supported by the findings of Verberckmoes et al. (2003) and Andersen et al. (2013), where a decreasing density of the cell layer was observed with an increasing concentration of strontium present in materials for bone regeneration. Strontium was introduced in the 1940s to tackle bone ailments as it can mimic calcium ions, and a significant reduction in fractures in osteoporotic patients was achieved. However, an increased concentration of strontium disturbs calcium deposition needed for the homeostasis of bone tissue (Alves Côrtes et al., 2024; Ricci et al., 2021). Concentrations from 5% lead to moderate-to-high toxicity, causing interference in the proliferation and differentiation of osteoblasts (Alves Côrtes et al., 2024). Strontium concentration also influences the pH-milieu in the extracellular matrix, with an increase in the pH to non-physiologic alkaline environment (Kruppke et al., 2020; Borciani et al., 2021; Shen et al., 2012). These results suggest a concentration-dependent cytotoxic effect of strontium, which was also observed by Schumacher et al. (2016). The positive effects of strontium toward cytocompatibility arise from its reduced concentration used in materials for bone regeneration, and thus, an optimum concentration has to be fixed for strontium while fabricating materials for bone regeneration (Ginebra et al., 2012; Hurtel-Lemaire et al., 2009). This necessitates deep research dwelling into the cytotoxic effects of strontium at various concentrations in cells making up the bone material, such as osteoblasts and MSCs.

In addition to the materials for bone regeneration, an investigation into the effects of BDNF addition to the cell culture media of MSCs derived from osteoporotic and non-osteoporotic subjects was performed. The studies demonstrated the positive effects of BDNF, leading to a reduction in both necrotic and apoptotic cell death in osteoporotic and non-osteoporotic MSCs. This reduction in necrosis and apoptosis was observed for all MSCs independent of the bone substitute material and the health status of the donors.

Limited research has been conducted on the role of BDNF in biomaterial-based bone regeneration. Zhang et al. (2017) and Zhang et al. (2020) demonstrated the role of BDNF in improving the migration of osteoblasts by regulating VEGF secretion. Previous studies were mainly focused on the presence and regulation of BDNF in bone fracture healing processes, and the direct effect of the molecule on bone MSC was minimally described (Kilian et al., 2014; Sandoval-Castellanos et al., 2021). Similar to other neurotrophins, BDNF exerts a regulatory effect on bone metabolism, exerting effects on the differentiation and proliferation of cells in the bone structure related to multiple myeloma (Liu et al., 2018; Brohlin et al., 2012; Su et al., 2018; Su et al., 2016). Feng et al. demonstrated that osteogenesis of bone marrow-derived MSCs was inhibited when an antisense RNA, BDNF-AS, was upregulated, plausibly because of the downregulation in the synthesis of BDNF; nevertheless, the study contradictorily shows the reduced proliferation of MSCs (Feng et al., 2018). In addition, Rezaee et al. (2010) demonstrated that BDNF leads to higher levels of the pro-inflammatory molecule interleukin 6 (IL-6), while Kauschke et al. (2018b) proved a reduction in pro-inflammatory molecules in the murine osteoporotic fracture model (Kauschke et al., 2018b; Rezaee et al., 2010). These opposing findings indicate the requirement for profound investigations on the beneficial effect of BDNF in bone MSC survival.

Moreover, some major studies have shed light on the role of BDNF in tissue regeneration in peripheral nerve regeneration, where the regulatory role and biomaterial-based delivery of the molecule for such applications were explored (McGregor and English, 2018). As clinical conditions involving fractures or defects are treated with bone biomaterials for the regeneration of lost or damaged bone tissue, an investigation into the interaction of such biomaterials and therapeutically relevant molecules in the bone microenvironment can foster the realm of bone regenerative medicine. Thus, this research work demonstrates one of the first such descriptions of the impact of BDNF on MSC survival at the interface of bone substitute biomaterials for bone regenerative applications during an osteoporotic clinical situation (Kilian et al., 2014; Kauschke et al., 2018b). Owing to the positive results, we intend to perform future preclinical studies in osteoporosis models. The current study, thus, serves as an initial path to obtain knowledge on the securing effect of BDNF against basic cell death programs of apoptosis and necrosis in MSCs when used with the PPGC+S material.

The combined positive effect of BDNF alongside PPGC+S 5:5 in securing the survival of MSCs by mitigating the apoptotic and necrotic cell death can be explored for designing biocompatible bone biomaterials for future applications. Thus, this study paves the way for further investigation into the role of BDNF and similar molecules in assisting the survival of bone-forming cells, such as MSCs and osteoblasts, over bone substitute materials.

## 5 Conclusion

In summary, this study reports a reduced rate of MSC necrosis with PPGC+S 5 + 5. The lowest apoptosis rate was measured for PPGC+S 3:7. Since it did not significantly differ from that of PPGC+S 5:5, we find that PPGC+S 5:5 is the most suitable ratio of calcium and strontium ion concentrations in pre-structured gelatin materials for bone regeneration. In addition, we showed for the first time that BDNF improves the survival of MSCs in combination with the tested materials for bone regeneration. These results imply the role of BDNF in the signaling pathway for apoptosis and necrosis. We, therefore, suggest the inclusion of BDNF in the pre-structured gelatin material of PPGC+S 5:5 for further *in vitro* and *in vivo* analyses. These findings support the research for evaluating specific materials and molecules for bone regeneration, especially for postmenopausal osteoporotic fractures.

## Data Availability

The raw data supporting the conclusions of this article will be made available by the authors, without undue reservation.
